# A Cross-National Comparison of Myalgic Encephalomyelitis and Chronic Fatigue Syndrome at Tertiary Care Settings from the US and Spain

**DOI:** 10.20448/801.51.104.115

**Published:** 2019-12-19

**Authors:** Shaun Bhatia, Nicole Olczyk, Leonard A. Jason, Jose Alegre, Judith Fuentes-Llanos, Jesus Castro-Marrero

**Affiliations:** 1 Center for Community Research, DePaul University, Chicago, IL, USA; 2 Center for Community Research, DePaul University, Chicago, IL, USA; 3 Center for Community Research, DePaul University, Chicago, IL, USA; 4 Vall d’Hebron University Hospital, CFS/ME Unit, Universitat Autònoma de Barcelona, Barcelona, Spain; 5 Vall d’Hebron University Hospital, CFS/ME Unit, Universitat Autònoma de Barcelona, Barcelona, Spain; 6 Vall d’Hebron University Hospital, CFS/ME Unit, Universitat Autònoma de Barcelona, Barcelona, Spain

**Keywords:** Myalgic encephalomyelitis, Chronic fatigue syndrome, Symptomatology, Cross-cultural comparison, Observational descriptive study, Functional impairment

## Abstract

Cross-national comparative studies are useful for describing the unique characteristics of complex illnesses, and can reveal culture-specific traits of disease frequency/severity and healthcare. Though myalgic encephalomyelitis (ME) and chronic fatigue syndrome (CFS) are debilitating conditions found all over the world, few studies have examined their characteristics across different countries. The purpose of this study was to compare the levels of functional impairment and symptomatology in patients with ME and CFS at tertiary referral hospitals in the US and Spain. Four hundred twenty potentially eligible participants (N = 235 from the US and N = 185 from Spain) who met the 1994 Fukuda et al. definition for CFS were enrolled. Both samples completed the medical outcomes study 36-item short-form health survey (SF-36) as a proxy for impairment, and the DePaul Symptom Questionnaire (DSQ) for patient symptomatology. ANCOVA and, where appropriate, MANCOVA tests were used to compare the SF-36 and DSQ items for illness characteristics between the samples. The patients from Spain demonstrated significantly worse functioning than those from the US in the SF-36 domains of physical functioning, bodily pain, general health functioning, role emotional, and mental health functioning. The Spanish sample also was also more symptomatic across all the DSQ-items, most significantly in the pain and neuroendocrine domains. These findings may be due to differences between the US and Spain regarding disability policy, perception of ME and CFS, and access to specialist care.

## 1. INTRODUCTION

Myalgic encephalomyelitis (ME) and chronic fatigue syndrome (CFS) are debilitating chronic, multi-systemic conditions of unknown origin, which are estimated to affect up to 24 million people worldwide (Johnston et al., 2013). ME and CFS is characterized by debilitating fatigue that last at least six months or more, and cannot be explained by any underlying medical condition. It can also include post-exertional malaise, memory and concentration problems, orthostatic intolerance, and unrefreshing sleep (Institute of Medicine, 2015).

Over the past 25 years, at least 20 case definitions have emerged for ME and CFS. (Brurberg et al., 2014) and individuals with ME and CFS have varied symptomatology dependent on the case definition used for diagnosis. This has created considerable challenges for researchers, as participants in different ME and CFS research studies may demonstrate variable symptom presentations. As an example of this problem, epidemiological studies have provided evidence suggesting that case definition operationalization captures different groups of individuals with ME and CFS (Jason et al., 1999; Reeves et al., 2007). In the United Kingdom (U.K.), Nacul et al. (2011) estimated a prevalence for ME and CFS of 0.19% when using only the Fukuda et al. (1994) definition, and a prevalence of 0.11% when using the Canadian Consensus Criteria (Carruthers et al., 2003).

With multiple case definitions, there exists an inherent potential for heterogeneity across research samples, which may limit researchers’ ability to replicate findings related to potential illness etiologies and presentation. Cross-cultural studies serve as exemplars highlighting the challenges associated with assessing symptomatic differences when using variant case definitions. In an international study of individuals with CFS referred to clinics in Germany, the US, and the UK, researchers reported no significant differences in impairment between the samples (Hardt et al., 2001). However, the authors noted that each sample was derived from operationalizing different case definitions, prompting speculation if the lack of observed symptomatic differences were an artifact of the case definitions. A similar study consisting of participants from clinics in Australia, the US, and the UK also reported no significant differences in impairment with the use of varying case definitions (Wilson et al., 2001).

The use of inconsistent ME and CFS case definitions has the potential to mask important population differences: for example, differences in access to specialist care and culturally-specific disease stigmatization. In addition, the lack agreement on a unified case definition leads to challenges in precisely describing the impairment and symptomatic presentation of the condition.

Given the aforementioned issues with sampling heterogeneity, there is a need to compare ME and CFS functional impairment and symptomatology in different countries using a consistent case definition. As an example, in one study that compared US and UK samples of patients with ME and CFS using the 1994 Fukuda case definition, the UK sample was significantly more impaired in terms of mental health and role emotional functioning, as well as specific symptoms of pain, neurocognitive difficulties, and immune manifestations (Zdunek et al., 2015). In addition, participants in the US sample reported more difficulties falling asleep, more frequently reported experiencing a sudden illness onset, and more often reported that the cause of illness was primarily due to physical causes.

The aim of this study was to compare a Spanish and US cohort of patients with ME and CFS using the same case definition. Additionally, patients in both samples were diagnosed by specialists in comparable tertiary care settings. The intent of these study control methods was to limit the scope of findings to differences of ME and CFS impairment and symptomatology that may be attributed to country. As this study was exploratory in nature, an emphasis was placed on investigating differences in ME and CFS patients across the two nations.

## 2. METHOD

### 2.1. Participants

#### 2.1.1. US Solve CFS BioBank sample

Data from the SolveCFS BioBank were shared with the DePaul University Research Team by the Chronic Fatigue and Immune Dysfunction Syndrome (CFIDS) Association of America. The SolveCFS BioBank has clinical information and blood samples on a cohort of English-speaking individuals (*n* = 242) aged over 18 years who were diagnosed by a US-based licensed physician specializing in CFS, ME/CFS, and ME using the 1994 Fukuda definition.

#### 2.1.2. Spanish Sample

Participants from the Spanish cohort came from an initial sample of 330 individuals recruited by a specialist physician with experience in diagnosing CFS and ME from a public tertiary referral center located in Barcelona, Spain. Individuals were surveyed using Research Electronic Data Capture (REDCap), a tool used for online data collection (Harris et al., 2009). Patients were eligible for the study if they were aged over 18 years and met the 1994 Fukuda definition.

### 2.2. Instruments

#### 2.2.1. Depaul Symptom Questionnaire (DSQ)

Participants from both samples completed the DePaul Symptom Questionnaire (DSQ), a self-report instrument used to assess ME and CFS symptomatology which includes a 54-item inventory covering the span of several ME and CFS symptom domains: fatigue, post-exertional malaise, sleep, pain, neurocognitive, autonomic, neuroendocrine, and immune (Jason and Sunnquist, 2018). Participants were asked to rate the frequency and severity of each symptom over the past six months on a five-point Likert scale. For each symptom, a composite score was generated by multiplying the frequency and severity symptom scores by 25 and averaging the sum, resulting in a 100-point scale. Higher scores were indicative of a higher level of symptomatology. The DSQ has been found to have good test-retest reliability as a standardized method to identify individuals with ME and CFS (Jason et al., 2015). It also has strong construct, convergent, and discriminant validity (Brown and Jason, 2014; Murdock et al., 2017).

The English version of the DSQ measure was translated into Spanish language by two experienced researchers, and retranslated by a professional native translator. The retranslated version was then discussed by the two researchers to reach a consensus on the developed version. The Spanish DSQ version has demonstrated very good psychometric properties in terms of test-retest reliability, and sensitivity and specificity (Jason and Sunnquist, 2018).

#### 2.2.2. Medical Outcomes Study 36-Item Short-Form Health Survey (SF-36 Or RAND Questionnaire)

The SF-36 is a short form self-report measure on functional status related to health (Sharpe, 1991). The SF-36 assesses functioning on eight subscales domains: physical functioning, role physical, bodily pain, general health, social functioning, vitality, role emotional, and mental health. Scores are ranged on a 0–100 scale, where a higher score indicates better functioning. The measure has been found to have good discriminant validity as a measure of mental health and physical functioning, and is both psychometrically and clinically valid in English and Spanish (McHorney et al,, 1993; Gandek et al,, 2004).

### 2.3. Procedure

#### 2.3.1. US Solve CFS BioBank Sample

Following approval by the DePaul University Institutional Review Board, participants were initially recruited by the CFIDS Association of America through their website, social networking devices, internet forums, and physician referral. All participants who met eligibility criteria completed a written informed consent process before being included in the sample. Participants completed the study measures by using REDCap or hard copy survey.

#### 2.3.2. Spanish Sample

Written informed consent was obtained from all participants before enrollment. The study protocol was approved by the local Vall d’Hebron University Hospital Institutional Review Board. Demographic and clinical data were recorded for each participant.

Spain is a member of the European Network on Myalgic Encephalomyelitis/Chronic Fatigue Syndrome (EUROMENE), which represents an interdisciplinary network of researchers emphasizing collaboration with the goal of improving quality of life among those with ME and CFS (European Cooperation in Science and Technology, 2015). With the inception of EUROMENE in 2016, several studies have been conducted exploring new challenges of biomedical research in individuals with ME and CFS from Spain (Castro-Marrero et al., 2017; Castro-Marrero et al,, 2017; Estèvez-López et al,, 2018). The current study is the first of a series of efforts to collaborate between EUROMENE and a group of researchers of ME and CFS from the US.

### 2.4. Statistical Analysis

Descriptive analysis of demographic characteristics for each cohort was performed. Linear hierarchical regression analyses based on one-way ANCOVA and, where appropriate, MANCOVA tests were used to analyze the data in the SF-36 domains and DSQ symptom items to compare illness characteristics between samples. Missing data were handled using pair-wise deletion. All calculated *p*-values were two-sided, and *p*-values < 0.05 were considered as statistically significant.

## 3. RESULTS

### 3.1. Description of Samples

#### 3.1.1. SolveCFS BioBank Sample

Of the original sample of 242 participants, 235 were included in the current study. Seven participants (2.9%) were excluded from analysis due to being under 18 years of age and exclusion as specified by the Fukuda case definition. The mean age of the sample was 50.1 years (*SD* = 12.4), and largely identified as female (73.6%) and White (98.3%). 57.9% of participants reported to be married or living with their partners, 58.6% reported to have a standard college degree or graduate degree, and 67.7% reported to be on disability.

#### 3.1.2. Spanish Sample

A total of 185 Spanish participants were included in the current study. From the original sample of 330 participants, 98 were not included (29.7%) due to psychiatric exclusionary conditions (e.g., major depression), other medical exclusions (e.g. hypothyroidism), and presence of lifelong fatigue – all specified as exclusions by the Fukuda case definition. An additional 47 participants were not included due to incomplete or missing demographic or DSQ data.

The mean age of the sample was 50.4 years (*SD* = 8.6). 84.9% identified as female, 98.9% were White, and 69.6% of participants reported to be married or living with their partners. The education status of the participants was fairly normally distributed, with the most people reporting to have some high school education (29.7%), and a high school degree or GED (25.9%). More than one-third of the sample (39.5%) reported to be on disability.

### 3.2. Demographics

The demographic characteristics for the US and Spanish samples are reported in Table 1. Univariate analysis of variance for the age of the samples indicated no significant difference [*F*(1, 408) = 20.62, *p* = 0.730]. Similarly, Pearson Chi-Square analysis indicated no significant difference in race between the samples [*χ^2^*(4,420) = 5.69, *p* = 0.223]. Significant differences were observed when examining the samples’ gender, marital, education, and work status distributions. Compared to the US sample, Spanish participants were more likely to be female [(*χ^2^*(1,420) = 7.78, *p* = 0.005] and married or living with a partner [(*χ^2^*(4, 416) = 19.11, *p* = 0.001], and were less likely to be on disability [*χ^2^*(6, 414) = 80.33, *p* < 0.001] and have higher levels of completed education [*χ^2^*(5,419) = 161.84, *p* < 0.001].

Based on these findings, subsequent analysis of functional status, and ME and CFS composite symptomatic scores were adjusted for gender, marital status, and education. Though group differences were observed for work status, this variable was omitted for analysis, as it was hypothesized to function as a byproduct of impairment rather than a predictive covariate. Binomial recodes for marital status (“Married or living with partner” or “Not married or living with partner”) and education status (“High school/GED or less” or “Some post-high school education”) were implemented for analyses to account for small or empty cell sizes based on the survey item responses Table 1. Group differences for both covariates were still found to be statistically significant after recoding.

### 3.3. Functional Status (SF-36)

Table 2 describes ANCOVA comparisons of the US and Spanish samples’ functional scores, adjusted for gender, marital status, and education. Higher scores on the SF-36 domains represent higher levels of functioning. The US sample had significantly better functioning than the Spanish sample in the domains of physical functioning [*F*(1, 414) = 7.39, *p* = 0.007], bodily pain [*F*(1, 414) = 59.68, *p* < 0.001], general health functioning [*F*(1, 415) = 4.38, *p* = 0.037], role emotional [*F*(1, 412) = 106.14, *p* < 0.001], and mental health [*F*(1, 414) = 113.04, *p* < 0.001]. Vitality among the Spanish sample was found to be moderately better than the US sample [*F*(1, 414) = 4.99, *p* = 0.026]. No differences of functioning were observed for the role physical and social functioning domains between the samples.

### 3.4. Composite Symptom Scores (DSQ)

The results of MANCOVA analyses of adjusted composite symptom domains and their respective embedded items are reported in Table 3. Higher symptom scores were indicative of higher levels of ME and CFS impairment.

With the exception of the single-item fatigue domain, all symptom domains demonstrated highly significant (*p* < 0.001) differences symptom based on the calculation of Wilks’ Lambda values. The symptom scores were higher in the Spanish sample for all 54 items, and in 50 of these items (93%), differences were found to be statistically significant (*p* < 0.05). The greatest adjusted mean differences between the samples were observed in the pain and neuroendocrine domains (average mean differences of items of 21.9 and 18.6, respectively).

## 4. DISCUSSION

Using consistent case definition and a validated ME and CFS instrument, our study suggests critical differences in the impairment levels experienced between the samples at tertiary care settings. It is possible that contextual features of Spanish healthcare and policy as they could pertain to adults with ME and CFS could help explain the impairment differences that were observed in this cross-cultural comparison.

Disability and employment status significantly differed between countries Table 1. The findings suggest that rather than accessing disability benefits, individuals in Spain with adverse health impairment may not be able to work and are unemployed, or are able to work, though presumably at a reduced capacity as they manage their illness. According to a 2010 report by the Organisation for Economic Co-operation and Development, Spanish people with disabilities face several challenges when attempting to reconcile the complexities of disability and employment: 1) Spanish labor law contain a series of obligations for employers, but these regulations are not enforced, 2) the disability benefit system needs to be adjusted to account for contemporary medical, economic, and labor market realities, and 3) individuals with disabilities tend to not readily sign up for employment programs because the system is too complex, comprised of decentralized and centralized actors (for example, the Public Employment Service, and the Institute of Social Security).

It is reasonable that the stress of working while managing ME and CFS might compound patient health and functioning. Being without federal assistance and employment may also contribute to greater severity of symptoms if managing the illness poses a financial burden on the individual. Differences in employment and disability between the samples could help account for the observed differences of functional impairment, where, in a majority of SF-36 domains, Spanish participants reported significantly lower health functioning compared to the US sample. Stigmatization of mental illnesses may play a role in the willingness or ability for individuals to receive treatment for mental health symptoms, and evidence suggests the existence of disparities of available mental health services in Spain compared to other European countries (Costa-Font et al., 2008). A lack of health professionals with specialization in ME and CFS in Spain may also contribute to the greater impairment of Spanish individuals in regards to their mental health functioning. Without proper understanding of the complexities of ME and CFS, certain symptoms may not be addressed properly.

Social functioning and the role of limitations due to physical health did not differ significantly between groups. Despite impairment in other domains, similar scores in these domains could be explained by cultural attitudes towards mental and physical health. Compared to other European countries, a lower proportion of people in Spain allow physical and mental health to impact their social activity (Costa-Font et al., 2008).

Similar to findings from the SF-36, ME and CFS symptom scores suggested more impairment in the Spanish sample. While levels of fatigue between groups were similar, Spanish participants reported significantly higher levels of impairment on a majority of the symptoms under each domain. This corroborates with the SF-36 outcomes, and suggests that parts of the current guidelines for management and treatment of ME and CFS in Spain need revision. Addressing this disparity in health and functioning might be due to cross-cultural differences between the countries. A large proportion of the Spanish participants are from Catalonia, which is worthy of consideration due to the decentralized nature of Spanish healthcare, and the inherent regional differences in healthcare policy. Between 2003 and 2014, Catalonia enjoyed the greatest increase in specialist care expenditure compared to the other autonomous communities in Spain (Nombela-Monterroso et al., 2018). However, Spain’s 2008 economic crisis caused drastic budget cuts which affected healthcare policy on both a national and regional level. A study involving health professionals in Catalonia examined the effects of these budget cuts on the quality of healthcare, and common themes included, but were not limited to, outdated technology, an increase in misdiagnoses, tired and overworked professionals, and human resource cutbacks (Porthé et al., 2018). Deteriorating quality of healthcare, the stigmatization of mental health, and the lack of ME and CFS specialization hinders the accessibility to proper care for patients with ME and CFS. An increase in physician’s education and specialization in ME and CFS is recommended; though addressing the many other issues facing the current state of healthcare in the region may also be prioritized to mitigate misdiagnosis and the use of outdated or insufficient treatment.

Patients from the US sample may benefit from better access to specialized healthcare professionals and advanced technology, but it is important to note that there are financial barriers which make healthcare less accessible for many in the US. Unlike Spain, health insurance in countries like the US is not guaranteed: approximately 9% of individuals in the US are uninsured (Berchick et al., 2018). Since healthcare costs are high and can cause financial insecurity, especially for those without access to insurance, many people may not receive adequate medical attention. Thus, while this sample of US individuals show less impairment than the Spanish patients, this may not be accurate for all living in the US with this illness. For those who lack access to regular medical care and medication, their impairment may be similar to those in the Spanish sample. This possibility is evidenced by one study that found variation in prevalence of fatigue amongst participants recruited from tertiary care facilities, primary care facilities, and community-based settings (Song et al., 1999). Additionally, patients receiving non-specialized care have been reported to be less satisfied with their level of care than those with access to a specialist (Sunnquist et al., 2017). Consequently, differences in impairment may be an effect of a lack of accessibility to specialized care and affordable healthcare, and improving accessibility to healthcare services could make diagnosis and treatment possible for more people with ME and CFS in the US.

### 4.1. Strengths and Limitations

This is the first collaborative study to compare the clinical features evaluated by the self-reported DSQ and SF-36 measures between US versus Spanish cohorts of individuals with ME and CFS.

An important factor in the ME and CFS field worth highlighting in this study is the lack of consensus on a case definition, and understanding the limitations of this deficiency. For instance, the Fukuda case definition used in this study has been criticized as being overly inclusive compared to other case definitions such as the Canadian Consensus Criteria (Carruthers et al., 2003) potentially including individuals who may not have ME and CFS, but instead have a psychiatric illness such as major depression (Institute of Medicine, 2015). As previously described, efforts were taken to not include participants in analysis who met various exclusionary health conditions, per the case definition. Notwithstanding, it is possible that the current study included patients with serious undiagnosed or unreported comorbidities. Psychiatric disorders, predominantly mood and anxiety disorders are a type of comorbidity commonly found in Spanish patients with ME and CFS (Castro-Marrero et al., 2017) which may partially explain the disparity in scores relating to mental health functioning and emotional well-being. Though a holistic understanding of ME and CFS should certainly account for highly prevalent comorbidities, including them in studies of impairment differences must be contextually acknowledged. Accounting for them in diagnosis and treatment could be a step towards improving the mental health and emotional well-being of Spain’s patients with ME and CFS.

The lack of specificity in the demographic data regarding employment status in the US sample is another constraint in this study, as better specification of the part-time and full-time employment categories could have bettered comparison. Also, this study sampled from patients already receiving tertiary care, which affects its generalizability.

### 4.2. Conclusions and Future Directions

Additional studies using comparable instruments and case definitions are needed to confirm the prevalence of symptoms and functional impairments in ME and CFS based on DSQ and SF-36 measures among other European, Latin-American, and the US populations. Furthermore, it would be valuable for future studies to account for the accessibility of healthcare when comparing patient symptomatology. Specifically, patients with ME and CFS report both geographic and financial barriers that limit their access to care, and these barriers in accessibility may vary between countries (Sunnquist et al., 2017). Adjusting for access to healthcare may mitigate some of the “funneling effect” occurring in individuals with a lack of options for specialized care winding up in a single treatment center, which may have led to a Spanish sample with fairly severe impairment. This adjustment could be made using a preexisting health index, such as the Healthcare Access and Quality Index developed by the Global Burden of Disease (2016) which provides accessibility metrics for the healthcare systems of 195 countries. Finally, future studies could create a multinational comparison of patients who may not be receiving tertiary care for their illness. This could broaden the understanding of differences in patient symptomatology beyond the scope of those who are already receiving specialized care.

## Figures and Tables

**Table-1.** Demographic characteristics of US and Spain samples.

Characteristic	US	Spain	*P*
	(*n* = 235)	(*n* = 185)	
	*M(SD)*	*M(SD)*	
Age (years)	50.1(12.4)	50.4(8.6)	.743
	%(n)	%(n)	
*Gender*			.005**
Male	26.4(62)	15.1(28)	
Female	73.6(173)	84.9(157)	
*Race*			.223
White	98.3(231)	98.9(183)	
Asian/Pacific Islander	0.9(2)	0.0(0)	
Black/African American	0.0(0)	0.5(1)	
American Indian/Alaska Native	0.0(0)	0.5(1)	
Other	0.9(2)	0.0(0)	
*Marital status*			.001**
Married or living with partner	57.9(136)	69.6(126)	
Separated	0.4(1)	3.3(6)	
Divorced	14.9(35)	14.4(26)	
Widowed	1.7(4)	2.2(4)	
Never married	25.1(59)	10.5(19)	
*Education*			< .001***
Less than high school	0.0(0)	10.3(19)	
Some high school	0.0(0)	29.7(55)	
High school or GED	11.1(26)	25.9(48)	
Partial college	30.3(71)	18.9(35)	
Standard college degree	43.2(101)	9.7(18)	
Graduate school	15.4(36)	5.4(10)	
*Work status*			< .001***
On disability	67.7(155)	39.5(73)	
Student	3.9(9)	0.0(0)	
Homemaker	5.7(13)	8.1(15)	
Retired	10.0(23)	5.4(10)	
Unemployed	1.7(4)	16.2(30)	
Working part-time^a^	0.0(0)	9.2(17)	
Working full-time^a^	10.9(25)	21.6(40)	

Note:

* p < .05;

** p < .01;

*** p < .001.

GED = Graduate Equivalency Degree.

a Part-time and full-time work specification not available for US participants who indicated that they were working.

**Table-2.** ANCOVA comparison of US and Spain samples on the 36-item short form health survey (SF-36) domain scores, adjusted for gender, marital status, and education.

SF-36 domains	US (*n* = 233)	Spain (*n* = 181)	*p*
	*M*	*M*	
Physical functioning	37.2	30.0	.007**
Role physical	4.1	1.3	.067
Bodily pain	45.0	23.9	< .001***
General health functioning	26.7	22.4	.037*
Vitality	15.1	18.7	.026*
Social functioning	29.8	30.4	.847
Role emotional	70.3	20.7	< .001***
Mental health functioning	67.5	44.8	< .001***

Note:

* p < .05;

** p < .01;

*** p < .001.

**Table-3.** MANCOVA comparison of US and Spain samples on the DePaul symptom questionnaire (DSQ) symptom scores composites, adjusted for gender, marital status, and education.

	US	Spain	P
	(n = 233)	(n = 180)	
	**M**	**M**	
Fatigue	80.1	83.8	.074
Post-Exertional malaise			< .001***
Dead, heavy feeling	71.9	80.8	.002**
Next day soreness	70.1	84.3	< .001***
Mental tiredness	59.1	78.1	< .001***
Minimum exercise makes you physically tired	75.3	81.4	.029*
Sick after mild activity	71.7	75.8	.178
Sleep			< .001***
Unrefreshed waking up	79.9	85.5	.019*
Nap daily	56.2	76.9	< .001***
Falling asleep	59.1	69.6	.004**
Staying asleep	54.3	70.2	< .001***
Waking up early	44.9	71.4	< .001***
Sleeping all day	18.6	24.9	.071
Pain			< .001***
Muscle pain	63.4	83.3	< .001***
Joint pain	56.6	80.5	< .001***
Eye pain	26.7	53.3	< .001***
Chest pain	26.2	50.1	< .001***
Bloating	40.1	50.7	.003**
Stomach pain	37.4	64.0	< .001***
Headaches	49.0	71.1	< .001***
Neurocognitive			< .001***
Muscle twitches	32.3	68.7	< .001***
Muscle weakness	64.3	74.9	.001**
Sensitivity to noise	59.4	74.2	< .001***
Sensitivity to bright lights	56.9	69.4	.001**
Problems remembering things	67.5	78.8	< .001***
Difficulty paying attention	56.9	78.6	< .001***
Difficulty expressing thoughts	63.5	74.9	< .001***
Difficulty understanding things	50.4	61.5	.001**
Can only focus on one thing at a time	61.3	79.2	< .001***
Unable to focus vision and/or attention	42.7	66.0	< .001***
Loss of depth perception	24.4	39.4	< .001***
Slowness of thought	56.3	71.2	< .001***
Absentmindedness or forgetfulness	60.2	73.6	< .001***
Autonomic			< .001***
Bladder problems	31.3	49.6	< .001***
Irritable bowel problems	43.9	64.5	< .001***
Nausea	34.4	41.2	.037*
Feeling unsteady on feet	39.1	63.2	< .001***
Shortness of breath	37.5	56.1	< .001***
Dizziness or fainting	36.5	43.6	.034*
Irregular heartbeats	27.2	50.9	< .001***
Neuroendocrine			< .001***
Losing or gaining weight without trying	35.8	52.6	< .001***
No appetite	21.2	39.0	< .001***
Sweating hands	12.1	27.6	< .001***
Night sweats	35.1	50.1	< .001***
Cold limbs	40.5	65.5	< .001***
Feeling chills or shivers	30.0	52.6	< .001***
Feeling hot or cold for no reason	43.8	63.9	< .001***
Feeling like you have a high temperature	28.0	50.8	< .001***
Feeling like you have a low temperature	23.5	40.5	< .001***
Alcohol intolerance	32.1	45.1	.008**
Immune			< .001***
Sore throat	34.5	57.4	< .001***
Tender/sore lymph nodes	41.0	41.7	.841
Fever	14.0	28.3	< .001***
Flu-like symptoms	45.9	60.8	< .001***
Smells, foods, meds or chemicals make you sick	46.8	62.5	< .001***

Note:

* p < .05;

** p < .01;

*** p < .001.

## Highlights of this paper

- Cross-cultural comparative studies serve as exemplars highlighting the challenges associated with assessing differences in symptoms, functional limitations, and work disability when using variant case definitions for ME and CFS.
- Using a single case definition, differences were observed on an array of symptoms and functional impairments from individuals with ME and CFS recruited at tertiary centers from the US and Spain.
- Disparity in ME and CFS symptomatology and impairment between the US and Spain may be attributed to differences in disability policy, perception of ME and CFS, and access to specialist care.
